# An Intelligent Prediction for Sports Industry Scale Based on Time Series Algorithm and Deep Learning

**DOI:** 10.1155/2022/9649825

**Published:** 2022-06-24

**Authors:** Hui Liang

**Affiliations:** School of Physical Education, Pingdingshan University, PingDingShan467000, China

## Abstract

Aiming at some existing issues in the sports industry, the existing model is optimized by deep learning and time series theory based on the relevant algorithm, and the scale of the sports industry is analyzed and predicted by the model. The results show the following: (1) Based on the single-step prediction of time series, MNTS structural algorithm can be used to describe and study the sports industry scale with the single factor, and the correlation fitting degree is high. (2) Curves of different evaluation methods can include parts linear stage and nonlinear stage according to the magnitude of change. (3) Seen from the optimization model in this paper, the proposed method can describe both global and local trends of data. (4) It can be seen from the prediction curve that the overall state of fluctuation indicates that time will have a great impact on the relevant scale of the sports industry. Compared with single-step prediction, the accuracy of multistep prediction is higher, and the multistep prediction model based on time series can well characterize and predict the scale of the sports industry. By using the relevant time algorithm, the sports industry scale can be predicted and analyzed so as to provide theoretical support for the formulation and implementation of relevant policies.

## 1. Introduction

Time series can be seen everywhere in our real life and has been widely studied and applied. Thi Thu-Hong et al. [1] proposed a method of large gap filling for time series data based on effective information. The dynamic time correlation algorithm is used to compare the subsequences, and the method is verified by relevant data. The method can well represent the correlation performance of data. In order to optimize the application prospect of transformation algorithm in the field of time series classification, Shu et al. [2] adopted short isometric shape transformation to simplify the calculation distance of algorithm length and provided the relevant theoretical basis of the algorithm on the basis of the research of this method, so that the accuracy of time series can be approximately lossless under certain preconditions. The superiority and correctness of the proposed algorithm are verified by relevant experiments. Niranjan et al. [3] studied the spatiotemporal changes of related phenological indicators by using time series data and related optimization algorithms. It is feasible to use MODIS data based on time series to analyze the long-term changes in structure. This study can provide theoretical support for effective monitoring of relevant data. In order to make one time series better reflect the state of another time series, Chen et al. [4] proposed a parallel solution on the Apache Spark platform, using the Streaming real-time computing module.

Time series prediction plays an important role in various fields of real life [5]. For the study of multichannel time series residual network block under training, Jessica et al. [6] used variance analysis and pair comparison to describe data, and the study showed that centralized research on environmental changes from the perspective of time series and spatial algorithms could better describe and characterize such typical recession phenomena and patterns. To study the optimization system based on time series and neural networks, Cheng et al. [7] provided the model by using the Internet of things and generalized neural network technology and realized remote data transmission by adopting modular computing. It is worth noting that the predicted results of the data are mainly obtained recursively through the correlation algorithm. Based on raw data from the global positioning network, Kaplan and Lau [8] analyzed structural data in different environments by using new high-resolution grid time series and lightning density climatology. To improve the optimization ability of complex data, Li et al. [9] proposed two methods to test statistics based on relevant theoretical analysis. Based on the theory of time series and neural networks, the cumulative Logistic regression model can be used to analyze the ordered time series. Based on this model, the consistency of change point estimation can also be studied by optimizing the relevant data, and a new method is proposed to estimate the location of multiple things [10, 11].

There are some problems in calculating the optimal interval length and proper weights of fuzzy time series, which will influence the varying aspects. To solve these problems existing in permutation entropy, Rostaghi and Azami [12] introduced dispersion entropy to quantify time series. In order to explore the vibration characteristics based on time series. The results show that dispersion entropy based on the time effect can accurately detect the changes in noise bandwidth, frequency, and amplitude. To explore the relationship between particulate matter, carbon monoxide, nitrogen dioxide, and other major pollutants and the respiratory system, Yang et al. [13] organically linked air pollutants with a variety of factors by using the generalized model of natural spline and principal component regression method based on relevant theories such as time series model and generalized neural network algorithm. Through the study of relevant cases, we can see that the relevant indicators show different changes in different regions. In a short monitoring period, air pollutants from traffic and the environment are related to the risk of respiratory infection. At the same time, the model can be used to predict the data and pollution emissions in the next ten years so that the optimization model based on time series can be well applied to environmental protection.

To predict the size of the sports industry, the time series based on single-step prediction and multistep prediction theory are introduced; they can optimize the memory network model to achieve an in-depth analysis of the scale of the sports industry and other related features. Based on the above discussions, the main contributions of this paper are given below:

1. In this paper, a sports industry scale prediction method combining time series and depth model is proposed to integrate the advantages of the two methods.
2. The proposed method can describe both global and local trends of sports data and achieve the single-step prediction and the multistep prediction results.

In this paper, in Section 1, we discussed some related papers and their achievements. In Section 2, we introduced some relevant theories to support this work. In Section 3, we propose an improved model based on time series and deep learning. In Section 4, we complete some relevant experiments using the improved model and also compare it to some baseline models. In Section 5, we conclude this paper and get some findings.

## 2. Relevant Theoretical Analysis

### 2.1. Time Series Prediction

(1) By setting the observed correlation time series as the measured values of the sensor, the corresponding trajectory matrix can be obtained as follows [14]:
  (1) $$\begin{array}{c} X^{n}={\left[X_{{}_{1}}^{n},X_{{}_{2}}^{n},\cdots ,X_{{}_{K}}^{n}\right]}_{L\times K}, \end{array}$$
where *Y*^*n*^ is time series; *X*^*n*^ is multidimensional sequence; *L* is embedded dimension; *K* is the relevant parameter.
(2) Through decomposition of the correlation singular value, it can be expressed as follows [15]:
  (2) $$\begin{array}{c} X^{n}=U_{L\times L}{\left[\begin{array}{c} \Delta 0\\ 00 \end{array}\right]}_{L\times K}V_{K\times K}^{T}, \end{array}$$
where △ is a positive singular value; *U* and *V* are left singular matrices and right singular matrices, respectively.
(3) The useful signal components of the sequence data are separated from the noise, and larger singular values are selected to describe the trajectory matrix as follows [16]:
  (3) $$\begin{array}{c} X^{n}\approx U_{L\times r}\Delta_{r\times r}V_{r\times K}^{T}={\left(h_{i,j}\right)}_{L\times K}, \end{array}$$
(4) By transforming the grouped matrix into the required length matrix, the relevant transformation formula is shown as follows:
  (4) $$\begin{array}{c} \left\{\begin{array}{c} y_{k}^{n}=\frac{1}{k}\sum_{m=1}^{k}h_{m}\left(1\leq k<L\text{p}\right),\\ y_{k}^{n}=\frac{1}{L_{\text{p}}}\sum_{m=1}^{L_{\text{p}}}h_{m}\left(L\text{p}\leq k\leq K\text{p}\right),\\ y_{k}^{n}=\frac{1}{T-k+1}\sum_{m=k-K\text{p}+1}^{T-k+1}h_{m}\left(K\text{p}<k<T\right). \end{array}\right) \end{array}$$

The calculation method of time series time-domain characteristics is shown in Table 1.

**Figure 1:**
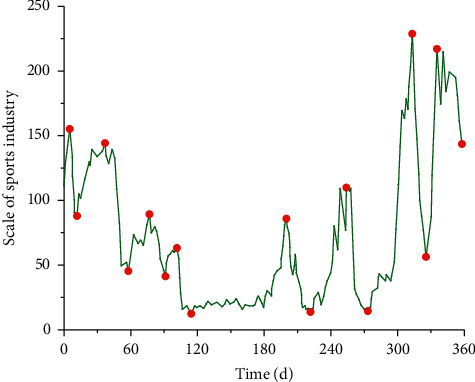
Changes of sports industry scale based on time series.

As a time-based structural algorithm, time series can monitor and analyze different data.  Figure 1 shows the one-year change curve of the sports industry scale in a certain region in 2020.

### 2.2. Deep Learning Model

**Figure 2:**
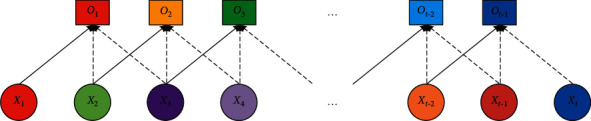
One-dimensional convolutional neural network.

Deep learning models are built on the working hierarchy when processing data and creating decision patterns. Since the traditional deep neural network cannot effectively solve the related problems, such as large errors, a deep neural network is used to find the related problems, such as time series classification. Wu et al. [17] proposed a hybrid convolutional neural network model combined with a genetic algorithm, which was based on the classification of time series of unreliable data based on a genetic algorithm. To obtain the optimal solution of the relevant grid, a genetic algorithm based on time series was designed, and the optimization way was verified by model calculation with actual data. Deep learning has been widely applied in digital organization and other related aspects [18]. Deep learning mainly predicts and analyzes relevant indicators of models by extracting relevant feature points of data. To identify digital organizations more accurately with neural network and deep learning, the model can be trained and verified on samples by quantifying the images of specific digital features and combining them with a quadratic programming method proposed. Wind speed prediction can optimize the industrial structure, which plays an indispensable role in product upgrading and transformation, and plays a crucial role in the optimization scheduling and system control of transformation. Chen et al. [19] proposed a nonlinear learning integration method applicable to wind energy based on relevant theories. This method uses a set of optimized structures to mine relevant information in different time series. Then the model realizes the optimization control of the related parameters of the top layer and forecasts and adjusts the wind speed by using the prediction system of the system. To verify the accuracy of the model in practice, several measured data were used to analyze and predict the wind speed. Time series model and prediction accuracy are the most difficult and challenging problems for data analysis and processing. To solve the existing related problems, Livieris et al. [20] proposed a complete optimization framework, which can be used to enhance the prediction range and prediction accuracy of the deep learning time series model. The framework mainly transforms the monitoring data under different time series to form high-precision time series data that can represent the original model. To test and verify the accuracy of the optimization model of time series data from different market segments for monitoring and analysis, the framework of the results of numerical experiments and statistical analysis can provide data for related theory research but also show that the optimization framework can significantly improve the deep learning model based on time series prediction performance. The one-dimensional neural network is shown in Figure 2.

#### 2.2.1. Residual Neural Network

The residual neural network can make use of its own advantages to alleviate the shortcomings of the convolutional neural network so as to ensure good prediction performance. Some parameters of the residual block are shown in Table 2 [21].

#### 2.2.2. Recursive Neural Network

The recursive neural network is a hot topic in deep learning research. RNN is a further extension of the traditional feed-forward neural network, which has the ability to process long-time sequence input. The research methods and steps of this neural network are as follows:

(a) The input gate is as follows [22]:
  (5) $$\begin{array}{c} i_{t}=\sigma \left(w_{i}\left[h_{t-1},x_{t}\right]+b_{i}\right). \end{array}$$
(b) The forgetting gate is shown below [23]:
  (6) $$\begin{array}{c} f_{t}=\sigma \left(w_{f}\left[h_{t-1},x_{t}\right]+b_{f}\right). \end{array}$$
(c) The output gate is as follows [24]:
  (7) $$\begin{array}{c} o_{t}=\sigma \left(w_{o}\left[h_{t-1},x_{t}\right]+b_{k}\right). \end{array}$$
(d) The update equation of the hidden state is as follows [25]:
  (8) $$\begin{array}{c} h_{t}=\tanh\left(c_{t}\right)*o_{t}, \end{array}$$

where *f*_*t*_, *i*_*t,*_ and *o*_*t*_ are forgetting gate, input gate, and output gate, respectively; wf, wi, and wo are weight matrices, respectively. *b*_*f*_, *b*_*i,*_ and *b*_*k*_ are bias terms; *h*_*t*-1_ and *x*_*t*_ are information and input information, respectively.

**Figure 3:**
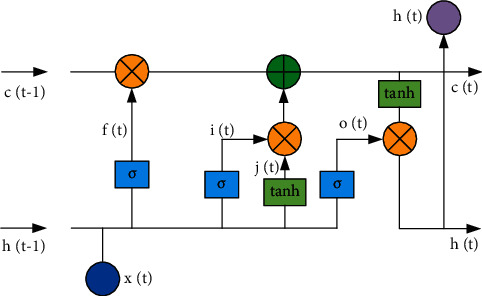
Structure diagram of LSTM.

As a new method, the neural network has obtained a series of applications in the fields of information theory and machine learning. However, there are some problems in the research process, including that the consistency of samples cannot be guaranteed when the samples between data are not independent. By confirming that the conditions and information between different neural estimators are consistent, it is shown that the probability of asymptotic convergence of neural network-based information estimators is 1. To detect anomalies in signal processing, neural network and Hilbert transform are used to construct a new optimization model based on deep learning and time series algorithm. Physiological health is very important for people's production and life. The theory of time series algorithm and neural network model can be used to analyze and predict physiological health. Firstly, a large number of monitoring means are used to monitor physiological data, and correlation algorithms are used to transform them into relevant signals that can be studied. Secondly, the modified time series algorithm and correlation model of deep learning are used to extract and analyze the signals. Relevant indicators can be obtained through analysis, and cases of physiological health can be further analyzed and processed through the classification of indicators. Finally, time series single-step and multistep analysis methods are used to predict so as to realize the application of physiological health and further provide theoretical support for individual safety. To better study the mechanism and characteristics in calculation and prediction, the method of structure diagram is adopted to describe the changes of the neural network, as shown in Figure 3.

## 3. Time Series Prediction Based on Memory Network

### 3.1. Time Series Single-Step Prediction

**Figure 4:**
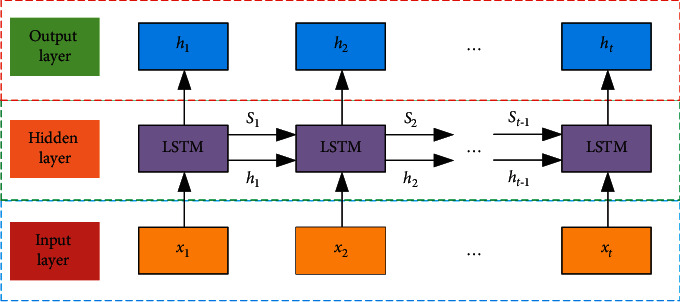
Global feature grid structure diagram.

Time series single-step prediction is used to predict the value of the next point in time. Time series is usually composed of multiple variables, and each of these factors affects the other, but people may only be interested in the change process of one of the variables. The structure of the global feature network in the LSTM network is shown in Figure 4.

By combining the weighted output vector with short-term historical data and inputting it into a full connection layer, the predicted value at time *t* can be obtained, as shown below [26, 27]:

(9) $$\begin{array}{c} y_{t}=W\left[u;O_{1};O_{2};\cdots O_{T};\right]+b. \end{array}$$

**Figure 5:**
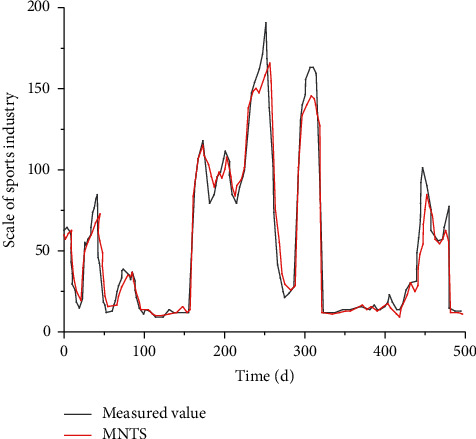
Comparison between measured values and MNTS.

To better illustrate the advantages and functions of MNTS, a statistical method is adopted to obtain the numerical value of the sports industry scale within 500 days in a certain region. Based on this data, the MNTS structural algorithm is adopted to calculate the sports industry, and the measured value is compared with the corresponding MNTS, as shown in Figure 5. It can be seen from the figure below that MNTS can describe and study the sports industry scale under single factor conditions, and the correlation fitting degree is relatively high.

### 3.2. Time Series Multistep Prediction

**Figure 6:**
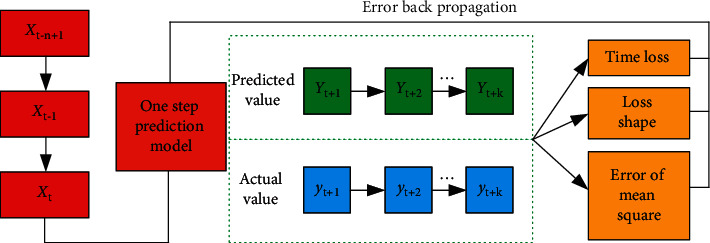
Structure diagram of the multistep prediction model.

Time series single-step prediction can only be used to predict the value of the next point in time; it is often useful to take corresponding measures according to the value multiple times, so the multistep prediction is needed to get the change of the monitored value of the system in the future period of time [28]. To give readers a better understanding of the proposed algorithm, the overall framework of the proposed algorithm is shown in Figure 6.

In this paper, a loss function is proposed based on different error measurements to evaluate the effect of multistep prediction.

(1) Shape loss is shown as follows [28]:
  (10) $$\begin{array}{c} D_{tw}\left(\hat{y_{i},}\overset{*}{y_{i}}\right)=\min\langle A,\Delta \left(\hat{y_{i},}\overset{*}{y_{i}}\right)\rangle , \end{array}$$
where *D*_*tw*_ is the dynamic time adjustment algorithm; *A* is the matrix sequence; $\hat{y_{i}}$ is the predicted value; $\overset{*}{y_{i}}$ is the true value.
(2) Time distortion loss is shown as follows [29]:
  (11) $$\begin{array}{c} T_{di}\left(\hat{y_{i},}\overset{*}{y_{i}}\right)=\langle \arg\min\langle A,\Delta \left(\hat{y_{i},}\overset{*}{y_{i}}\right)\rangle ,\Omega \rangle , \end{array}$$
where *T*_*di*_ is time distortion loss; Ω is the cost matrix.
(3) The mean square error is shown as follows [30, 31]:
  (12) $$\begin{array}{c} M_{se}\left(\hat{y_{i},}\overset{*}{y_{i}}\right)=\frac{1}{n}\sum_{i=1}^{n}{\left(y_{t}^{i}-\hat{y_{t}^{i}}\right)}^{2}, \end{array}$$

where *M*_*se*_ is the mean square error; $\hat{y_{t}^{i}}$ is the predicted value; *y*_*t*_^*i*^ is the true value; *n* is the number of predicted values.

Therefore, when setting the value of the prediction step *k*, related parameter settings under different evaluation indicators are shown in Table 3.

**Figure 7:**
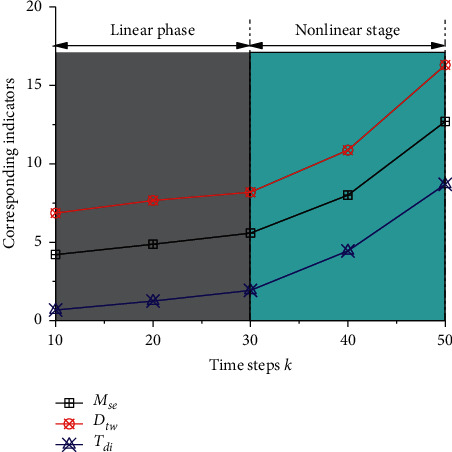
Index changes under different methods.

By analyzing the changes of different evaluation indexes under different time steps, the index change curves under different methods were obtained, as shown in Figure 7.

(1)As the number of time steps increases, indicators of different evaluation methods show a trend of gradual increase; (2) The curve shows an approximately linear change with a relatively small change range in the low step; when the number of time steps increases gradually, the curve shows a nonlinear change with a large change range. According to the amplitude of change, the curve can include the linear and nonlinear stages, and the number of time steps at the cut-off point is 30. (3) From a quantitative point of view, the index values of different evaluation methods are different, among which the corresponding index of *D*_*tw*_ is the largest, the corresponding index of *M*_*se*_ is relatively low, and the corresponding index of *T*_*di*_ is the smallest in the whole evaluation index.

## 4. Research on Sports Industry Estimation

### 4.1. Introduction to Sports Industry

After decades of reform and opening up, China's sports have made great progress and entered a stage of stable development. Especially the 2008 Beijing Summer Olympic Games ushered in a new stage of development for Chinese people's sports and made the concept of national fitness deeply rooted in people's hearts. The 2022 Beijing Winter Olympics has given China a new opportunity for the development of snow and ice sports. However, there are also some problems in the development process [32–34]:

1. Firstly, the national fitness service system needs to be improved. With the process of population urbanization, more and more people flood into cities, which poses new challenges to the internal fitness service system of cities. At the same time, in the vast rural areas, the construction of a basic fitness service system still needs considerable input of manpower and material resources.
2. Secondly, basic sports such as “three big balls“ are still weak. China has certain advantages in table tennis, diving, and other sports, but from the perspective of volleyball, basketball, and football, China's voice in the international arena needs to be further improved.
3. Finally, the sports industry market is not competitive enough. China's sports market and China's sports cause complement each other. Sports markets can promote the creativity of the sports market to a certain extent. However, the Chinese sports market has been monopolized by well-known foreign brands for a long time, which has restricted the development of Chinese domestic brands for a long time, thus restricting the development of the sports industry.

Therefore, although China's sports industry is developing very rapidly, it still faces many challenges in the development process. It is necessary to use the method based on time series to predict the scale of the sports industry so as to better serve the sports market in China.

### 4.2. Forecast Contents and Results

The total size of the sports industry in a certain place in the past 20 years was collected as the original data for corresponding modeling analysis. The relevant original data sequence established based on the time series model is as follows [35]:

(13) $$\begin{array}{c} X_{\left(0\right)}=\left(x_{\left(0\right)}^{1},x_{\left(0\right)}^{2},x_{\left(0\right)}^{3},\cdots ,x_{\left(0\right)}^{n}\right),\\ X_{\left(1\right)}=\left(x_{\left(1\right)}^{1},x_{\left(1\right)}^{2},x_{\left(1\right)}^{3},\cdots ,x_{\left(1\right)}^{n}\right). \end{array}$$

There are limitations of a single accuracy standard, so three indexes *Mae*, *Mre,* and *Rmse* are the index as follows [36]:

(14) $$\begin{array}{c} M_{ae}=\frac{1}{m}\sum_{i=1}^{m}\left|y^{\left(i\right)}-y{'}^{\left(i\right)}\right|,\\ M_{re}=\frac{1}{m}\sum_{i=1}^{m}\left|\frac{y^{\left(i\right)}-y{'}^{\left(i\right)}}{y^{\left(i\right)}}\right|,\\ R_{mse}=\sqrt{\frac{1}{m}\sum_{i=1}^{m}{\left(y^{\left(i\right)}-y{'}^{\left(i\right)}\right)}^{2}}. \end{array}$$

**Figure 8:**
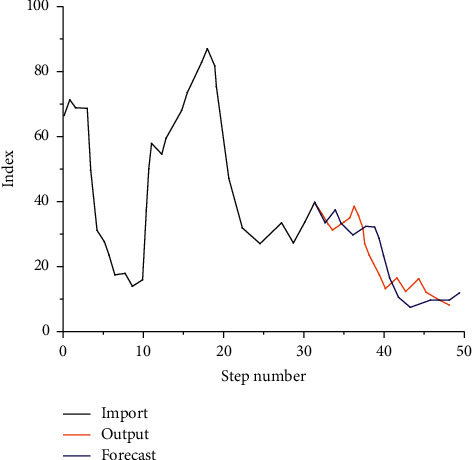
Statistical prediction results.

**Figure 9:**
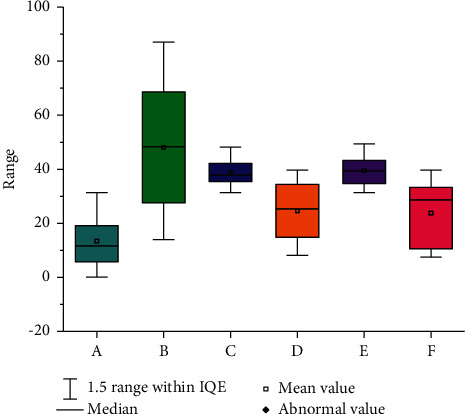
Data boxplot.

By using an iterative algorithm to analyze the scale of the sports industry, the prediction results of relevant statistical data are as in Figures 8 and 9.

**Figure 10:**
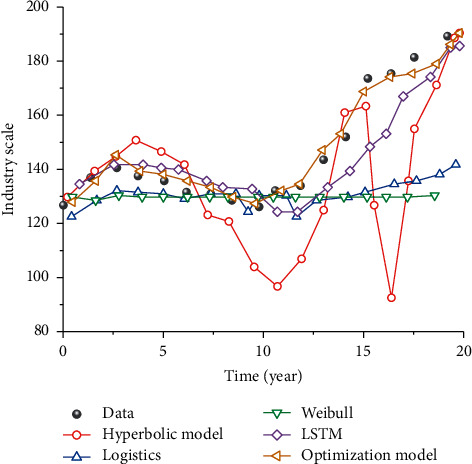
Comparison of curves of different models.

Meanwhile, to test the correlation accuracy of the evaluation index, the relevant evaluation criteria are selected as shown in Table 4:

The corresponding curves of different models are shown in Figure 10: (1) It can be seen from the hyperbolic model that the model can well represent the first rise change, but it is relatively poor in describing the decline of the industrial scale and has a great change from the actual data. (2) From the perspective of Logistics, the change of this model is mainly shown as a slow rise, then keeps constant, and finally reaches a change curve of slow rise. This model is suitable for describing the situation where the data change is not much, and the description of the data fluctuation is relatively poor. (3) From the Weibull distribution model, the overall performance of this model is an approximately horizontal curve, and the correlation with experimental data is poor. (4) From the perspective of the LSTM distribution model, this model can well represent the overall trend of data changes. In the first stage, it can well represent the changes in relevant data, but in the later stage, there are some deviations in the description of curves. On the whole, the description is very good compared with the above model. (5) Seen from the optimization model in this paper, it can not only well describe the overall change form of data but also well represent each stage of data. It shows that the model can well describe the scale of the sports industry at different times.

Relevant evaluation of the sports industry scale under different indicators and models is obtained by using the above methods, as shown in Table 5.

### 4.3. Research on Sports Industry Scale Prediction

**Figure 11:**
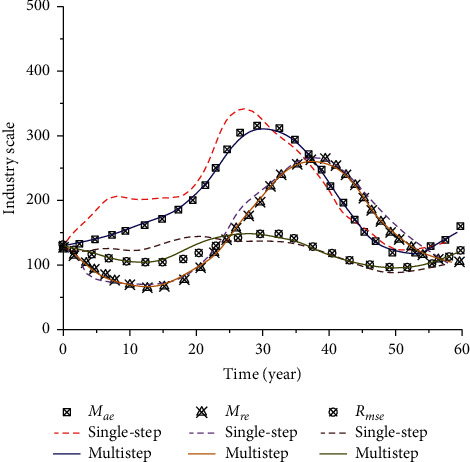
Industry forecast chart under different time series.

Through the prediction and analysis of the relevant scale of the sports industry by different time series algorithms, the model can well describe and characterize the relevant scale of the sports industry. The ultimate purpose of using algorithms to analyze related structures is to predict and analyze the feature points of related structures so as to guide the following research through the prediction results. Therefore, to further analyze and predict the relevant contents of the sports industry scale, a single-step prediction of time series is adopted to analyze the above relevant sports industry scale in the following 60 years, as shown in Figure 11.

By adopting single-step prediction and multistep prediction, it can be seen that with the gradual increase of time, the size of the sports industry presents different trends: (1) From the perspective of *M*_*ae*_, as time goes by, the industrial scale first slowly increases. When it reaches 30 years, the industrial scale increases to the peak, and then the curve gradually decreases as time goes by. When it reaches 53 years, the curve reaches the new lowest value. (2) From the perspective of *M*_*re*_, the increase of time makes the scale of the sports industry decline gradually at first and then increase slowly. After reaching the peak, the curve continues to decline until the end. (3) From the perspective of *R*_*mse*_, the variation range of this curve is relatively small compared with the above two situations, showing a state of fluctuation on the whole, indicating that time will have a great impact on the relevant scale of the sports industry. In addition, it can be seen from the figure above that compared with single-step prediction, the accuracy of multistep prediction has been improved, indicating that the time series can well represent and predict the scale of the sports industry and provide theoretical support for the formulation and implementation of relevant policies.

## 5. Conclusion

In this paper, time series is adopted to research and study the sports industry scale, and an optimization model is used to predict the industrial scale. The relevant conclusions are as follows:

1. On the basis of statistical data, MNTS structural algorithm is adopted to analyze and study the sports industry, indicating that the MNTS algorithm can well predict and study the size of the sports industry under the condition of a single factor.
2. The curve presents an approximate linear change in the low curve; the curve presents a nonlinear change in the increasing steps, and the corresponding change range becomes larger and larger.
3. It can be seen from the corresponding curves of different models that compared with the Hyperbola model, Logistics model, Weibull model, and LSTM model, the optimization model in this paper can not only well describe the overall change form of data but also well represent each stage of data.
4. Industry forecast under different time series shows that compared with the prediction that is relatively accurate, the multistep prediction model based on time series can well represent and predict the size of the sports industry, providing theoretical support for the formulation and implementation of relevant policies.

## Figures and Tables

**Table 1:** The time-domain characteristics of the sample sequences.

Time-domain feature	Index	Computing method
Trend	*T* trend	Regression is performed on the moving average
Seasonal fluctuation	*S* season	Observations in the same phase during different periods
Autocorrelation	Huest index	Using heavy range regression to find *H*
Chaos	Punovo exponent	Iterative ratio solution

**Table 2:** The parameters of residual neural network.

Residualblock sequence	The volumeof the convolutionkernel	Number ofconvolutionkernels	Windowsize
1	16	32	1
2	16	32	2
3	16	64	1
4	16	64	2
5	16	128	1
6	16	128	2
7	16	256	1
8	16	256	2

**Table 3:** Residual neural network parameters.

Evaluation index	*K*				
	10	15	20	25	30
*M*_*se*_	4.21	4.87	5.58	8.00	12.7
*D*_*tw*_	6.86	7.66	8.19	10.87	16.3
*T*_*di*_	0.68	1.25	1.93	4.45	8.69

**Table 4:** Residual neural network parameters.

Index	Accuracy requirement			
	Excellent	High	Middle	Low
The checker ratio *Q*_1_	＜0.2	＜0.4	＜0.5	＜0.7
Probability of error *Q*_2_	＞0.8	＞0.7	＞0.6	≤0.6
Grey correlation *Q*_3_	＞0.9	＞0.8	＞0.7	＞0.6

**Table 5:** Industrial scale prediction evaluation.

Index	Hyperbolic model	Logistics model	Weibull model	LSTM	Optimization model
*M*_*ae*_	1.30	0.16	0.63	3.11	0.78
*M*_*re*_	0.72	0.23	0.48	0.48	0.35
*R*_*mse*_	0.79	4.05	1.28	0.15	0.16
*Q*_1_	1.26	2.38	1.36	0.67	0.54
*Q*_2_	2.15	2.75	0.04	0.42	1.48
*Q*_3_	0.79	2.36	0.17	0.18	2.16

## Data Availability

The data used to support the findings of this study are available from the corresponding author upon request.
